# A Test for Pre-Adapted Phenotypic Plasticity in the Invasive Tree *Acer negundo* L.

**DOI:** 10.1371/journal.pone.0074239

**Published:** 2013-09-09

**Authors:** Laurent J. Lamarque, Annabel J. Porté, Camille Eymeric, Jean-Baptiste Lasnier, Christopher J. Lortie, Sylvain Delzon

**Affiliations:** 1 Department of Biology, York University, Toronto, Ontario, Canada; 2 University of Bordeaux, UMR 1202 BIOGECO, Talence, France; 3 INRA, UMR 1202 BIOGECO, Cestas, France; CNRS, Université de Bourgogne, France

## Abstract

Phenotypic plasticity is a key mechanism associated with the spread of exotic plants and previous studies have found that invasive species are generally more plastic than co-occurring species. Comparatively, the evolution of phenotypic plasticity in plant invasion has received less attention, and in particular, the genetic basis of plasticity is largely unexamined. Native from North America, *Acer negundo* L. is aggressively impacting the riparian forests of southern and eastern Europe thanks to higher plasticity relative to co-occurring native species. We therefore tested here whether invasive populations have evolved increased plasticity since introduction. The performance of 1152 seedlings from 8 native and 8 invasive populations was compared in response to nutrient availability. Irrespective of nutrients, invasive populations had higher growth and greater allocation to above-ground biomass relative to their native conspecifics. More importantly, invasive genotypes did not show increased plasticity in any of the 20 traits examined. This result suggests that the high magnitude of plasticity to nutrient variation of invasive seedlings might be pre-adapted in the native range. Invasiveness of *A*. *negundo* could be explained by higher mean values of traits due to genetic differentiation rather than by evolution of increased plasticity.

## Introduction

Phenotypic plasticity has been widely recognized as an important feature for plants to cope with environmental changes [1], [2]. Numerous studies have shown that plants are plastic for a large array of traits related to structure, development, metabolic activity, morphology, physiology, phenology, and reproduction [3]–[10]. Phenotypic plasticity has also been classified as a major determinant of the success of invasive species by increasing fitness relative to native species in recipient communities [11], [12], [13]. Broader distributions of alien species are correlated with higher levels of plasticity in response to increasing resource availability [14], and on artificial gradients, invasive species are also more plastic than co-occurring native or non-invasive species [15]–[20]. Davidson et al. [21] recently synthesized this work via a meta-analysis of 75 pairs of invasive/native species concluding that invasive species do express greater phenotypic plasticity than native species irrespective of the response traits measured. However, there are instances that did not support this pattern [22]–[25], and it has been suggested that the success and fitness advantage of invasive species can be mediated by the expression of constant higher mean trait values across different environments and not necessarily by the plasticity of these traits [26]–[29]. For instance, invasive species frequently possess higher trait values for growth rate [30], [31], [32], lower leaf mass per area [30], [33], [34], and advanced leaf unfolding and flowering periods [35], [36], [37]. Phenotypic plasticity should therefore be considered in combination with mean trait values when testing for plant invasiveness.

Higher plasticity levels of invasive species are generally hypothesized to be related to post-introduction evolution of phenotypic plasticity [13], [38], [39]. In this regard, intraspecific contrasts across environmental gradients have been analyzed in the following cases: (i) between populations from native and invasive ranges [40], [41] and (ii) between populations within the invasive range [12], [42], [43]. Overall, no general pattern has emerged to date. Invasive populations of *Senecio inaequidens* were for instance more plastic than their native conspecifics in response to fertilization [44] while no difference was observed between native and invasive populations of *Microstegium vimineum* across a large array of environments [45]. Variation in light and soil moisture availability induced differences in plasticity for above-ground biomass and leaf mass per area among invasive populations of *Microstegium vimineum* but not for reproduction-related traits among invasive populations of *Polygonum cespitosum* [42], [43]. Pre-adapted plasticity might therefore be a common feature of several invasive plants, and it should now be more explicitly tested.

A total of 357 tree species has been reported to be invasive worldwide disrupting major native ecosystem structure and functioning [46], [47], [48]. Invasive trees are thus appropriate models to evaluate the role of ecological and evolutionary processes in invasion given their large impacts, frequency, and longevity [49]–[53]. To date, most studies examining the importance of phenotypic plasticity in tree invasion compared invasive *vs.* native tree species [15], [39], [54], [55]. With the exception of *Melaleuca quinquenervia* and *Triadica sebifera* [39], [56], the genetic differences in plasticity between native and invasive populations of exotic trees are unexamined.

Porté et al. [57] recently found that the invasive tree *Acer negundo* significantly expressed higher magnitude of phenotypic plasticity than its co-occurring native species with increasing environmental resources, and particularly nutrient availability. The purpose of this study was therefore to examine the genetic basis of plasticity in *A*. *negundo*, *i.e.* to determine whether higher plasticity of invasive populations is due to post-introduction evolution or pre-adaptation in the native range. The performance of native and invasive populations of *A*. *negundo* was compared across a gradient of nutrient availability. Life-history traits related to growth, physiology, leaf morphology and biomass and known to promote plant invasiveness [31], [32] were measured. We hypothesize that invasive populations possess greater plasticity in growth and associated traits relative to populations from the native range. These findings would support the idea that plasticity could have evolved in the introduced range. In contrast, the absence of difference in plasticity between populations from native and invasive ranges would indicate that higher plasticity of invasive populations of *A*. *negundo* could be due to pre-adaptation in the native range.

## Materials and Methods

### Studied Species

*Acer negundo* L. (Box Elder or Manitoba maple) is a widely distributed mid-successional species native to North America. Its distribution range extends from southern Alberta and central Manitoba to Mexico and Guatemala southward and from central Montana to New England states and central Florida eastward [58], [59], [60]. This species is frequently found in floodplains and riparian habitats but can also occur in dry coniferous forests, oak savannas, and grasslands [61], [62]. *A*. *negundo* was intentionally introduced in the Old Continent at the end of the seventeenth century, *i.e.* in 1688 in England, and in France in 1749 [60], [63]. Currently, *A*. *negundo* is highly invasive throughout southern, central and eastern Europe [60], [64]. It frequently occurs not only in riparian habitats characterized by high rate of flood disturbance and high soil nutrient level [65], [66] but also under drier conditions along roadsides, industrial wastelands, and dry ruderal sites [52], [67].

### Experimental Design

Seeds of *A*. *negundo* were harvested between September and November 2009 from eight native populations sampled in Ontario and Quebec, Canada and from eight invasive populations located in the Landes and Gironde departments of Aquitaine region, Southern France (Table 1). No specific permissions were required for these locations that are not part of protected areas and do not involve endangered species. All native and invasive populations were sampled from riparian forests. Populations in the invasive range were distributed within the Adour-Garonne river basin. Seeds came from 9 to 12 maternal trees in each source populations with maternal trees randomly selected and at least 10 m apart. In February 2010, 30 seeds per maternal tree were subjected to a cold treatment (14 weeks at 5°C in a cold chamber) at the INRA research station of Pierroton, France (44°44′N, 0°46′W). In spring 2010, 27 seeds per maternal tree were sown into 4 L (15×15×17.7 cm) pots filled with a commercial sphagnum peat soil mixture (organic matter 80%, pH = 6). We first sowed three seeds per pot until germination and then kept one seedling in each pot thereby generating a total of 90 seedlings per source population. Pots were then placed under a greenhouse that was side-opened to permit wind and insects to enter. We did not control light and temperature that approximated ambient conditions. Seedlings were watered twice a week to saturation.

**Table 1:** The 16 source populations sampled to examine phenotypic plasticity of invasive tree species *Acer negundo* L.

Province/Department	Collection site	River	Latitude	Longitude	Distance to thenearest population (km)
**Native range**					
Ontario	Paris	Grand	43°12′27″N	80°21′58″W	65
Ontario	Fergus	Grand	43°41′53″N	80°22′50″W	65
Ontario	Nicolston	Nottawasaga	44°10′40″N	79°49′02″W	18
Ontario	Angus	Nottawasaga	44°18′59″N	79°53′08″W	18
Ontario	Toronto Home Smith park	Humber	43°39′06″N	79°29′44″W	26
Ontario	Toronto Serena Gundy park	Don	43°43′05″N	79°21′15″W	26
Quebec	Sherbrooke	Saint-François	45°23′44″N	71°52′50″W	24
Quebec	Windsor	Saint-François	45°34′04″N	72°00′23″W	24
**Invasive range**					
Landes	Saubusse	Adour	43°39′22″N	01°11′13″W	10
Landes	Riviere-Saas-et-Gourby	Adour	43°40′29″N	01°08′06″W	10
Landes	Pontonx-sur-l’Adour	Adour	43°47′03″N	00°55′30″W	35
Gironde	Cestas	Eau Bourde	44°45′20″N	00°40′49″W	30
Gironde	Bruges	Les Jalles	44°54′13″N	00°36′16″W	30
Gironde	Moulon	Dordogne	44°51′30″N	00°13′10″W	19
Gironde	Castillon-la-Bataille	Dordogne	44°51′04″N	00°02′16″W	19
Gironde	St-Denis-de-Pile	Isle	44°59′34″N	00°12′29″W	22

A split-plot design was used with nutrient level as the fixed main effect and range of *A*. *negundo* populations (native or invasive) as the fixed sub-effect with all native and invasive populations subjected to three nutrient levels. We selected 72 seedlings from 8 to 10 families (*i.e.* maternal trees) per population for a total of 1152 seedlings structured as follows: 6 blocks × 3 nutrient levels × 2 ranges × 8 populations × 4 individuals. The experiment was initiated on February 17^th^, 2011 and lasted 147 days. Nutrients were applied on the 25^th^, 53^th^, 81^st^ and 109^th^ days of the experiment. The nutrient treatment corresponded to the addition of the complete slow release 16-7-15 (NPK plus micronutrients) fertiliser Floranid Permanent (Compo France SAS, Levallois-Perret, France). In the low nutrient level (N0), seedlings did not receive any additional fertilizer. In the medium and high nutrient levels (N1 and N2, respectively), seedlings received four fertilizer doses equivalent to 0.125 g and 0.500 g N each, for a total of 0.500 g and 2 g N, respectively. The high nutrient level corresponded to the nutrient availability encountered by *A*. *negundo* populations in soils of the invaded riparian habitats of southern France [68], [69]. A previous study conducted *in situ* also showed that invasive individuals of *A*. *negundo* had a leaf N content averaging 1.17 gN.m^−2^ [57]. The N0 and N1 treatments thus represent levels of nutrient that are below the average field conditions in the introduced range.

### Gas Exchange

Photosynthetic rate measurements were performed on 192 seedlings. In each treatment, four individuals from different families and blocks were randomly sampled per source population. The measurements were done on sunny days between June 20^th^ and July 7^th^. Leaf gas exchange measurements were carried out with a portable steady-state flow-through chamber (PLC6) connected to an infrared gas analyser (CIRAS-2, PP Systems, Hitchin, UK) equipped with temperature, humidity, light and CO_2_ control modules. Net gas exchanges were measured within a sealed cuvette of 2.5 cm^2^, with an air CO_2_ concentration of 380±3 ppm, a temperature of 22±0.5°C and a relative humidity of 80±10% of ambient, controlled by regulating the flow diverted through a desiccant. To obtain the maximum assimilation rate per unit leaf area (*A*_area_, µmol CO_2_.m^−^^2^.s^−1^) at ambient CO_2_, leaves were illuminated with a red-blue light source attached to the gas exchange system and maintained at saturated light (PPFD = 1500 µmol PAR.m^−2^.s^−1^). Prior to the measurements, the gas analyser was calibrated in the laboratory using 400 ppm standard gas, while full CO_2_ and H_2_O zero and differential calibrations were performed in the field after each set of six measurements. Up to three measurements were carried out on each sampled individual, and data were recorded when assimilation curves remained stable for more than 20 s. All measurements were taken between 8.00 and 11.00 solar time on fully expanded and sun-exposed leaves to avoid midday stomatal closure.

### Leaf Morphology and Biochemistry

Leaf nitrogen content and morphological traits were measured on 288 seedlings representing six individuals per population and per treatment (including those used for gas exchange measurements). Leaves were sampled on the same days as the photosynthetic rate measurements. Three to five leaves were collected per sampled individual. Leaf surface area was measured with a planimeter (Light Box model, Gatehouse, Scientific Instruments LTD, Norfolk, UK) and the average leaf size (L_s_, cm^2^) was calculated. Leaves were then placed in an oven at 65°C until constant dry weight and leaf dry mass was later weighed with an electronic weighing scale (Explorer Pro, EP 114 model, Ohaus Corporation, Pine Brook, NJ, USA). Leaf mass per area index (LMA, g leaf.m^−2^ leaf) was calculated as the ratio of leaf weight by leaf area. Finally, leaf samples were crushed to a powder with a ball mill (MM 200, Fisher Bioblock Scientific, France) and leaf nitrogen content (N_mass_, %) was determined using an elementary analyser Eager 300 CHNOS (FlashEA 1112, ThermoElectron Corporation, Waltham, MA, USA). The maximum assimilation rate per unit leaf mass (*A*_mass_, µmol CO_2_.g^−1^.s^−1^) was calculated as the *A*_area_ to LMA ratio, the leaf nitrogen content per leaf area (N_area_, g N.m^−2^) as the product of N_mass_ and LMA, and the photosynthetic N-use efficiency (PNUE, µmol CO_2_.g^−1^ N.s^−1^) as the *A*_area_ to N_area_ ratio.

### Growth and Biomass

A total of seven individuals died during the course of the experiment and therefore, final height and stem collar diameter of 1145 seedlings were recorded on July 4^th^. A graduated pole to 0.01 m accuracy was used to record heights, and diameters were measured with an electronic calliper to 0.01 mm accuracy. The 288 individuals previously used for morphological measurements were harvested on July 14^th^ after 147 days of growth. Above-ground biomass was separated into stems and leaves, and roots were separated from soil and washed. Biomass was oven-dried at 65°C until constant dry weight and further weighed using an electronic weighing scale (Explorer Pro, EP 114 model, Ohaus Corporation, Pine Brook, NJ, USA). The following traits were calculated: total biomass (*W*_t_, g), above-ground biomass (*W*_a_, g), leaf biomass (*W*_l_, g), stem biomass (*W*_s_, g), root biomass (*W*_r_, g), total leaf area (*A*_l_, m^2^), root:shoot ratio (RSR, g.g^−1^), leaf weight ratio (LWR, g leaf.g^−1^ plant), stem weight ratio (SWR, g stem.g^−1^ plant), root weight ratio (RWR, g root.g^−1^ plant) and leaf area ratio (LAR, m^2^ leaf.g^−1 ^leaf).

### Statistical Analyses

Differences in traits were tested with a generalized linear mixed model that was fit to a split-plot design (procedure MIXED, REML method in SAS, version 9.2, SAS Institute, Cary, NC, USA) [70]. We used nutrient level, range, and the interaction of nutrient level × range as fixed factors whilst block, block × range, population nested within range, and the interaction of nutrient level × population nested within range were treated as random factors. To account for the influence of plant size on biomass allocation [71], we used total biomass as a covariate when we tested the following traits: *A*_l_, RSR, LWR, SWR, RWR and LAR. Type III sums of squares were used for the calculation of *F* statistics. Random effects were further evaluated using a log likelihood ratio (LLR) test from the full and reduced models. All factors were identified significant at alpha <0.05. A significant range effect for a given trait indicated an overall genetic differentiation between seedlings from native and invasive populations. Moreover, phenotypic plasticity was examined here at the population-level [13], [72]. A significant effect of nutrient level indicated plasticity for a given trait. The difference in plasticity of a given trait between seedlings from native and invasive populations was reported when the interaction of nutrient level × range was significant. The variation of trait of native and invasive seedlings was also reported as follows: (1−(trait_env2/_trait_env1_))*100. Lastly, we calculated the Relative Distance Plasticity Index (RDPI) [73], and the Plasticity Index (PI) [5] for two experimental nutrient level changes, low-to-medium and medium-to-high, as follows:



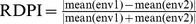
.



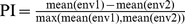
.

For each trait, the two indexes were calculated for each population using mean values in each treatment (*i.e.* nutrient level). The difference in RDPI and PI between native and invasive ranges was examined using a generalized linear mixed model with range as a fixed factor and population nested within range as a random factor.

## Results

### Overall Trends

**Figure 1:**
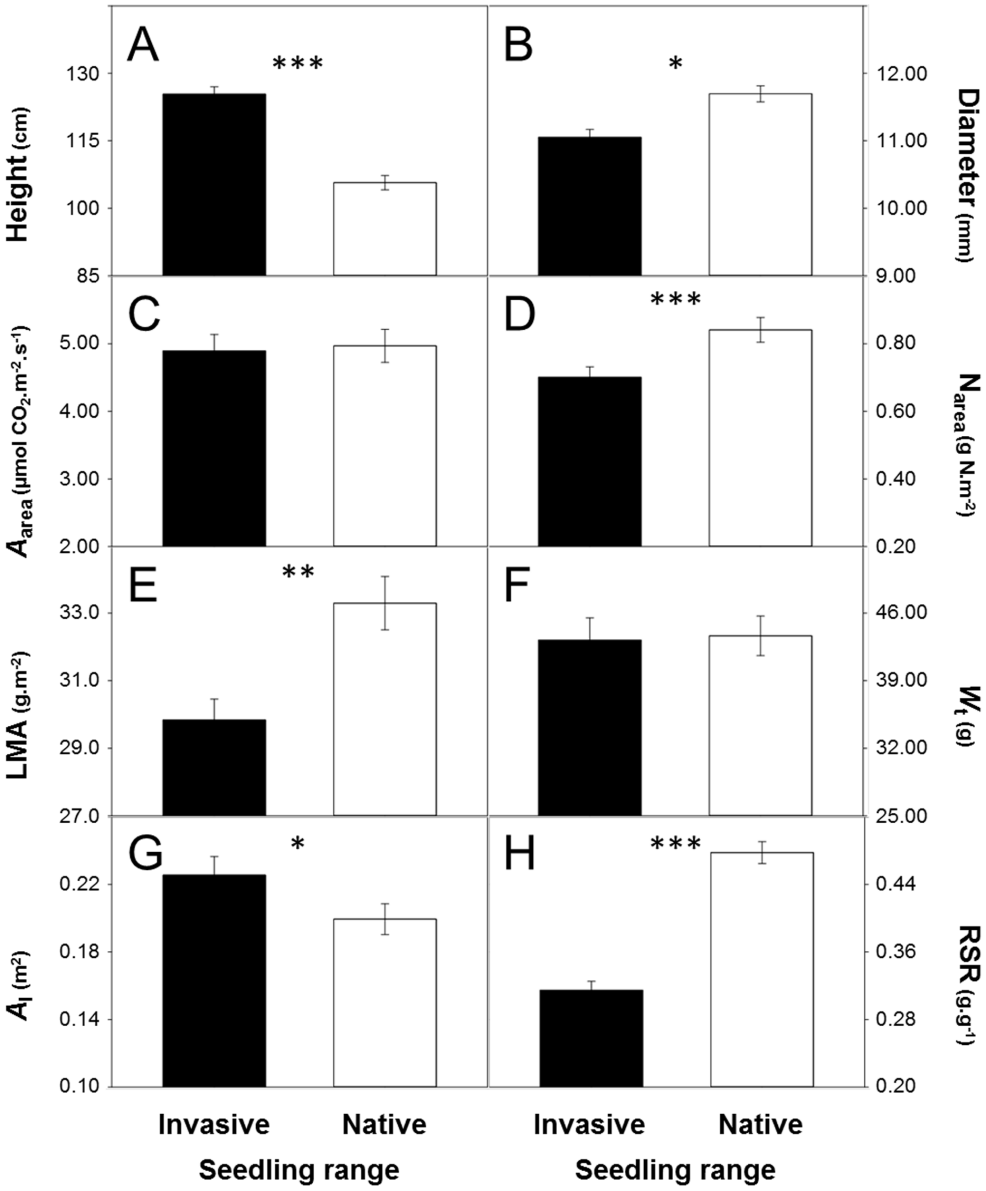
Mean ± SE of life-history traits for native and invasive seedlings of *Acer negundo*. Differences in growth (A,B), physiology (C,D), leaf morphology (E), biomass (F,G) and biomass allocation (H) were calculated across nutrient levels. n = 576 (height and diameter), 96 (*A*_area_) and 144 (N_area_, LMA, *W*_t_ and *A*_l_) per range. See text for definition of terms. * *P*<0.05, ** *P*<0.01, *** *P*<0.001.

Irrespective of nutrients, individuals of *A*. *negundo* from invasive populations expressed significantly greater heights and smaller diameters than their native conspecifics (significant range effect; Table 2; Fig. 1A, B; see Table S1 for means per treatment). There was no significant difference in maximum assimilation rates (*A*_area_ and *A*_mass_; Fig. 1C), and invasive seedlings had lower leaf nitrogen contents (N_area_ and N_mass_; Fig. 1D) and greater PNUE (Table 2). Invasive seedlings also had lower average leaf size and LMA (Table 2; Fig. 1E). There were no statistical differences in total and aboveground biomass (Table 2; Fig. 1F). Seedlings from invasive populations however allocated more resources to foliage than to roots, displaying greater *A*_l_, LWR, SWR and LAR, and lower *W*_r_, RSR and RWR compared to seedlings from native populations (Table 2; Fig. 1G, H). Significant genetic variations were found in height among invasive populations (within invasive range: LLR = 5.6, *P* = 0.018; within native range: LLR = 0.6, *P* = 0.44) and in diameter among native populations (within native range: LLR = 14.1, *P* = 0.0002; within invasive range: LLR = 0.5, *P* = 0.44).

**Table 2:** Generalized linear mixed models (GLMM) analyses of traits related to growth, gas exchange and leaf morphology, biomass and biomass allocation in eight native and eight invasive populations of *Acer negundo* along a nutrient gradient.

		Source of variation									
		Nutrient		Range		Nutrient × range		Population (range)		Nutrient × population (range)	
Traits	AIC	*F*	*P*	*F*	*P*	*F*	*P*	LLR	*P*	LLR	*P*
**Growth**											
Diameter (mm)	5237.0	55.93	**<.0001**	5.92	**0.0289**	0.14	0.8718	12.3	**0.0004**	2.9	*0.0885*
Height (cm)	11004.7	39.43	**<.0001**	39.33	**<.0001**	2.37	0.1123	5.3	**0.0213**	4.2	**0.0404**
**Leaf traits**											
*A*_area_ (µmol CO_2_.m^−2^.s^−1^)	784.7	38.09	**<.0001**	0.04	0.8392	0.00	0.9980	2.7	0.1003	5.2	**0.0226**
*A*_mass_ (µmol CO_2_.g^−1^.s^−1^)	−467.9	31.78	**<.0001**	0.06	0.8098	0.24	0.7876	0.3	0.5839	0.9	0.3428
N_area_ (g N.m^−2^)	1.9	116.61	**<.0001**	28.96	**<.0001**	2.95	*0.0543*	0.0	1	0.0	1
N_mass_ (%)	486.0	194.18	**<.0001**	7.58	**0.0087**	0.13	0.8772	0.5	0.4795	2.8	*0.0943*
PNUE (µmol CO_2_.g^−1 ^N.s^−1^)	929.2	17.91	**<.0001**	2.86	0.1135	0.03	0.9748	2.8	*0.0943*	0.6	0.4386
LMA (g.m^−2^)	2032.0	0.97	0.4069	8.94	**0.0098**	0.10	0.9087	0.7	0.4028	1.1	0.2943
L_s_ (cm^2^)	2343.6	14.44	**<.0001**	20.21	**0.0005**	0.31	0.7393	0.8	0.3711	0.2	0.6547
**Biomass**											
*W*_t_ (g)	2590.9	17.06	**<.0001**	0.02	0.8985	0.23	0.7928	0.0	1	0.9	0.3428
*W*_a_ (g)	2454.3	18.93	**0.0002**	1.24	0.2709	0.25	0.7803	0.0	1	1.5	0.2207
*W*_l_ (g)	1559.5	37.29	**<.0001**	0.16	0.6915	0.11	0.8944	0.0	1	1.0	0.3173
*W*_s_ (g)	2338.2	14.51	**0.0006**	1.60	0.2132	0.35	0.7099	0.0	1	1.5	0.2207
*W*_r_ (g)	1886.0	9.47	**0.0001**	10.74	**0.0059**	0.24	0.7837	0.0	1	1.1	0.2943
*A*_l_ (m^2^)	−609.1	14.42	**0.0012**	5.62	**0.0326**	1.45	0.2355	1.3	0.2542	0.5	0.4795
**Biomass allocation**											
RSR (g.g^−1^)	−308.2	20.25	**<.0001**	54.33	**<.0001**	0.38	0.6846	0.7	0.4028	3.0	*0.0833*
LWR (g leaf.g^−1^ plant)	−1011.7	32.35	**<.0001**	5.22	**0.0277**	1.74	0.1893	0.7	0.4028	0.7	0.4028
SWR (g stem.g^−1^ plant)	−772.7	0.06	0.9401	66.33	**<.0001**	2.43	0.1068	1.6	0.2060	4.2	**0.0404**
RWR (g root.g^−1^ plant)	−705.8	17.63	**<.0001**	53.89	**<.0001**	0.11	0.8943	1.3	0.2542	3.7	*0.0544*
LAR (m^2^ leaf.g^−1^ leaf)	−2577.3	29.76	**<.0001**	9.40	**0.0083**	0.79	0.4631	0.1	0.7518	2.9	*0.0886*

*F* values are given for fixed effects while log likelihood ratios (LLR) are given for random effects. Statistically significant values (*P*<0.05) are shown in bold and marginally significant values (*P*<0.1) are shown in italic. The Akaike Information Criterion (AIC) value of the model used is given for each variable. See text for definition of terms.

### Trait Plasticity

**Figure 2:**
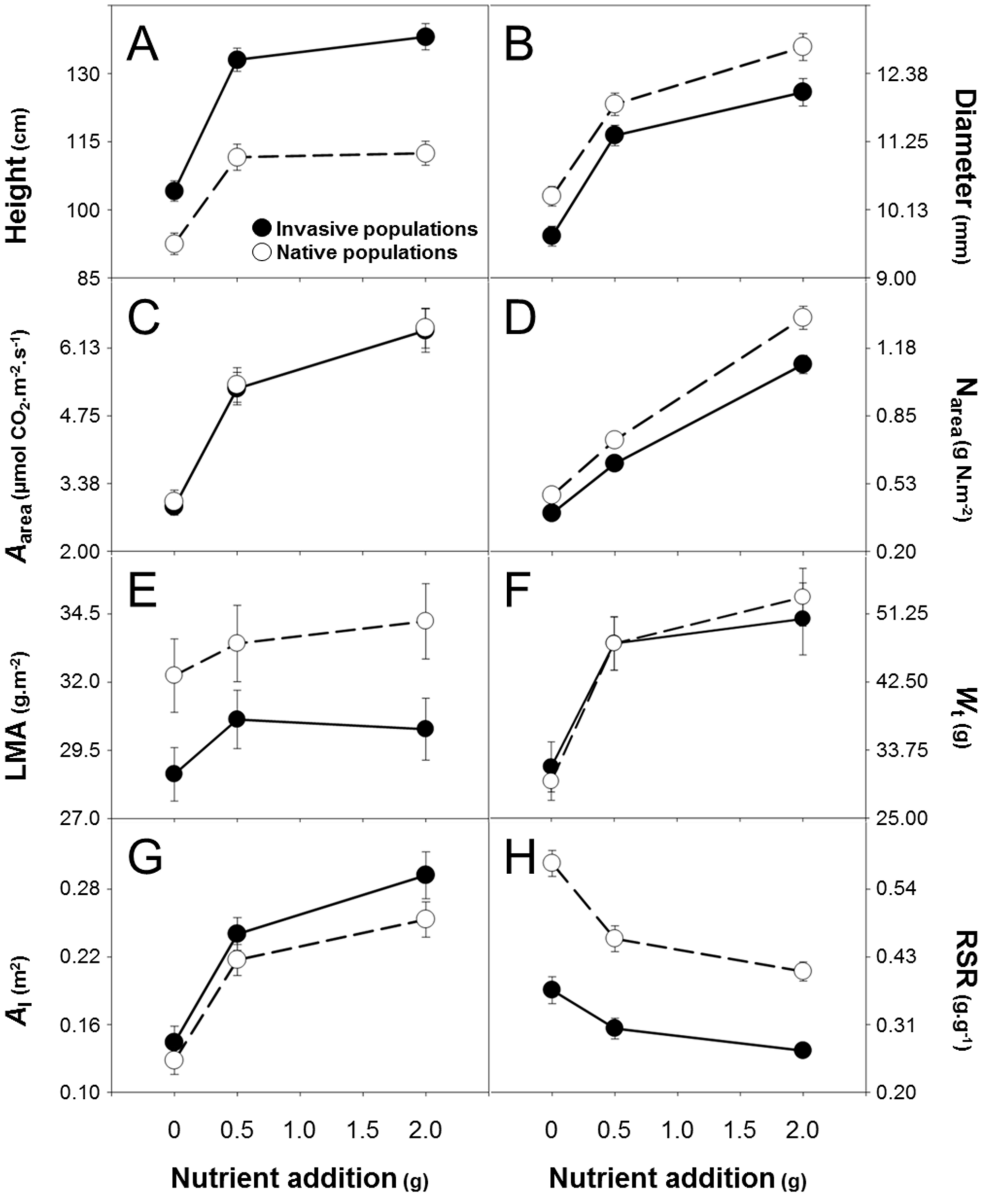
Plasticity of native and invasive seedlings of *Acer negundo* to nutrient availability. Means ± SE of traits related to growth (A,B), physiology (C,D), leaf morphology (E), biomass (F,G) and biomass allocation (H) are represented. n = 192 (height and diameter), 32 (*A*_area_) and 48 (N_area_, LMA, *W*_t_ and *A*_l_) per range and nutrient level. See text for definition of terms.

Seedlings of *A*. *negundo* responded significantly to increases in nutrients (significant nutrient effect for all traits but LMA and SWR; Table 2; Fig. 2) with increased growth, maximum assimilation rate, total biomass, and above-ground allocation (AGB, TLA, LWR, LAR) and decreased below-ground allocation (RSR, RWR). The change from low-to-medium nutrient conditions had a stronger effect on seedling trait values than the change from medium-to-high nutrient conditions. Individuals of *A*. *negundo* respectively showed a 19%, 44% and 35% increase in height, maximum assimilation rate and total biomass from low-to-medium nutrient conditions but a 2%, 18% and 9% increase from medium-to-high nutrient conditions (Fig. 2; see Tables 3 and 4 for trait RDPI and PI values). Across all populations, traits such as SWR and LMA showed low plasticity along the nutrient availability gradient (mean RDPI_SWR_ = 0.03, mean RDPI_LMA_ = 0.07) while *W*_l_, *A*_area_ and N_area_ exhibited larger changes (mean RDPI*_W_*_l_ = 0.21, mean RDPI*_A_*_area_ = 0.22, mean RDPI_Narea_ = 0.25).

**Table 3:** Relative Distance Plasticity Index (RDPI) along a nutrient gradient for populations of *Acer negundo* from the native and invasive ranges.

	RDPI low-to-medium nutrient levels		RDPI medium-to-high nutrient levels	
Traits	Invasive	Native	Invasive	Native
**Growth**				
Height	0.12±0.02	0.10±0.02	0.04±0.01	0.05±0.01
Diameter	0.08±0.01	0.07±0.01	0.04±0.01	0.04±0.01
**Leaf traits**				
*A*_area_	0.29±0.04	0.28±0.04	0.19±0.03	0.14±0.04
*A*_mass_	0.24±0.04	0.25±0.06	0.15±0.03	0.11±0.03
N_area_	0.24±0.03	0.22±0.02	0.27±0.02	0.28±0.04
N_mass_	0.20±0.04	0.19±0.03	0.27±0.02	0.25±0.03
PNUE	0.11±0.04	0.14±0.04	0.21±0.05	0.22±0.04
LMA	0.06±0.02	0.09±0.02	0.03±0.01	0.10±0.02*
L_s_	0.11±0.01	0.07±0.03	0.11±0.03	0.08±0.02
**Biomass**				
*W*_t_	0.23±0.04	0.26±0.06	0.08±0.02	0.14±0.04
*W*_a_	0.24±0.04	0.28±0.07	0.09±0.03	0.15±0.05
*W*_l_	0.28±0.06	0.31±0.08	0.12±0.04	0.15±0.04
*W*_s_	0.23±0.04	0.28±0.07	0.09±0.02	0.16±0.05
*W*_r_	0.21±0.05	0.20±0.05	0.09±0.04	0.11±0.03
*A*_l_	0.27±0.06	0.28±0.08	0.11±0.03	0.10±0.02
**Biomass allocation**				
RSR	0.15±0.03	0.12±0.04	0.09±0.03	0.10±0.01
LWR	0.11±0.04	0.09±0.02	0.08±0.04	0.06±0.04
SWR	0.04±0.01	0.04±0.01	0.02±0.01	0.03±0.01
RWR	0.11±0.02	0.09±0.03	0.07±0.02	0.07±0.01
LAR	0.13±0.04	0.13±0.03	0.12±0.03	0.16±0.03

Comparisons of RDPI using a Generalized Linear Mixed Model with range as a fixed factor and population nested within range as a random factor. Significant difference between ranges (*P*<0.05) denoted by an asterisk. See text for definition of terms.

**Table 4:** Plasticity Index (PI; Valladares et al. 2000) along a nutrient gradient for populations of *Acer negundo* from the native and invasive ranges.

	PI low-to-medium nutrient levels		PI medium-to-high nutrient levels	
Traits	Invasive	Native	Invasive	Native
**Growth**				
Height	−0.22±0.03	−0.17±0.03	−0.04±0.03	−0.01±0.04
Diameter	−0.14±0.02	−0.13±0.02	−0.06±0.03	−0.07±0.02
**Leaf traits**				
*A*_area_	−0.44±0.04	−0.43±0.05	−0.14±0.11	−0.14±0.09
*A*_mass_	−0.38±0.04	−0.38±0.09	−0.13±0.10	−0.07±0.08
N_area_	−0.38±0.03	−0.35±0.03	−0.43±0.02	−0.43±0.05
N_mass_	−0.31±0.07	−0.32±0.05	−0.43±0.02	−0.39±0.04
PNUE	−0.14±0.07	−0.17±0.09	0.33±0.07	0.35±0.06
LMA	−0.06±0.05	−0.02±0.07	0.01±0.03	−0.02±0.08
L_s_	−0.12±0.06	−0.08±0.07	−0.17±0.05	−0.09±0.05
**Biomass**				
*W*_t_	−0.30±0.09	−0.35±0.10	−0.07±0.06	−0.07±0.10
*W*_a_	−0.32±0.10	−0.39±0.09	−0.10±0.06	−0.10±0.11
*W*_l_	−0.40±0.08	−0.43±0.08	−0.21±0.06	−0.13±0.10
*W*_s_	−0.29±0.10	−0.37±0.10	−0.07±0.06	−0.09±0.12
*W*_r_	−0.23±0.11	−0.28±0.09	−0.01±0.08	0.00±0.08
*A*_l_	−0.40±0.08	−0.39±0.09	−0.18±0.06	−0.13±0.05
**Biomass allocation**				
RSR	0.14±0.09	0.20±0.06	0.09±0.06	0.11±0.06
LWR	−0.18±0.06	−0.17±0.03	−0.13±0.04	−0.06±0.04
SWR	0.00±0.03	−0.05±0.03	0.01±0.02	−0.02±0.03
RWR	0.12±0.07	0.14±0.05	0.06±0.05	0.07±0.04
LAR	−0.12±0.09	−0.12±0.08	−0.15±0.07	−0.03±0.11

Comparisons of PI using a Generalized Linear Mixed Model with range as a fixed factor and population nested within range as a random factor. See text for definition of terms.

There were no significant differences in plasticity between seedlings from native and invasive populations for any traits (non-significant nutrient × range effect; Table 2; Fig. 2). There was also no difference in RDPI or PI for any traits but the RDPI_LMA_ between medium and high nutrient levels did differ (Tables 3 and 4; across the whole gradient, mean trait RDPI = 0.15 and 0.14 and mean trait PI = −0.14 and −0.15 for native and invasive populations, respectively). The magnitude of plasticity differed at the population level for height, maximum assimilation rate, and SWR (significant nutrient × population effect; Table 2).

## Discussion

Higher magnitudes of plasticity relative to native species are common in invasive plants, particularly in invasive trees [15], [21], [74]. Nevertheless, these differences are not necessarily a product of post-introduction evolution and can also be explained by innate characteristics. This null hypothesis was tested and supported in this study using the highly invasive tree species *Acer negundo*. Although increased nutrient availability is a key component of tree recruitment dynamics [75], [76], this artificial gradient tested here did not elicit differences in plasticity between native and invasive seedlings. Pre-adapted plasticity to nutrient availability is thus a reasonable explanation for the successful spread of this species, at least at this early stage of development.

The evolution of plasticity in invasive species is relatively infrequent and no consensus has been reached in the literature so far (Table S2). Variation in resource conditions lead to differences in plasticity between seedlings from native and invasive populations for perennials *Centaurea stoebe* and *Taraxacum officinale* and trees *Melaleuca quinquenervia* and *Triadica sebifera* [39], [56], [77], [78] but not for the annual grass *Microstegium vimineum*, the biennnial forb *Alliaria petiolata* and the perennial shrub *Clidemia hirta* [40], [45], [79]. However, a rigorous assessment of the origin and importance of plasticity in plant invasion requires both inter- and intraspecific contrats [39]. In response to nutrient availability, invasive seedlings of *A*. *negundo*, which had shown increased plasticity relative to than their co-occurring native species across the same resource gradient [57], expressed here similar response for all life-history traits compared to their native conspecifics. Our results therefore reflect innate characteristics of plasticity that would be pre-adapted in the native range. This supports the outcome observed for *Triadica sebifera* in response to water availability: invasive seedlings exhibited greater growth than seedlings of native *Schizachyrium scoparium* but not than their native conspecifics [39]. The only other study that conducted both inter- and intraspecific comparisons across the same resource gradient did not find any difference in plasticity to CO_2_ enrichment between native and invasive populations of *Eupatorium adenopherum* and the native congener *Eupatorium japonicum* [80].

Seedlings from native and invasive populations of *A*. *negundo* significantly differed in most of their traits across the gradient of nutrient availability. Invasive seedlings consistently exhibited higher values for traits associated with invasiveness, *i.e.* higher growth rate, lower LMA, and greater allocation to foliage [30], [34]. This supports many other studies which posit that genetically-based advantages in plant size and above-ground biomass for invasive over native genotypes may promote the success of invasive species [81]–[84]. For instance, invasive individuals of *Melaleuca quinquenervia* and *Triadica sebifera* also outperformed native congeners [39], [56], [85]. Interestingly, invasive seedlings of *A*. *negundo* did not achieve greater height growth via physiological advantages but only via a preferential allocation to foliage. Significant lower leaf nitrogen content and similar maximum assimilation rate were found here. This contradicts recent studies on the genetically-based difference of functional traits in invasive plant species that showed higher values of physiological traits for invasive genotypes [86], [87], [88]. These divergences might be due to the rapid adaptation of *A*. *negundo* in its introduced range reflecting a change in adaptive strategy. Whilst plasticity may not have evolved *de novo*, it is possible that most of the traits conferring faster growth (such as greater allocation to above-ground biomass) may have done so to provide a competitive advantage over native species of recipient communities.

Multi-species comparisons in the native range of exotic plant species showed that invasive aliens differed in traits but not in plasticity from their non-invasive alien congeners [26], [28], and pre-adaptation of plasticity in invasive plant species might finally be more common than expected. Phenotypic plasticity is a common denominator for invasive plant species but tolerance of invasive genotypes across a broad range of conditions might rely more on a combination of life-history traits rather than on evolved plasticity in the introduced range. This would be the case for *A*. *negundo* since the species occupies wide and similar ranges of habitats such as wet-rich and dry-poor nutrient riparian forests both in North America and in Europe [52], [61], [89]. Furthermore, various mechanisms such as founder effects, multiple introductions, and selective pressures can drive genetic differentiation between native and invasive populations. Molecular analyses using neutral markers over large areas sampled including whole native and invasive ranges would thus be necessary to fully understand the role of these factors [90]. Given that there was no consistent variation in traits amongst populations from the invasive range, genetic data would provide valuable information on the origin of those populations sampled in French riparian areas, *e.g.* whether they have all undergone similar selective pressures or come from the same pool of native populations which were not sampled in this study (*i.e.* founder effects).

## Conclusions

The origin of increased plasticity in invasive plant species is an important and relatively understudied set of hypotheses. Given the geographical scope of the populations we were able to sample herein, pre-adaptation is a more viable explanation for the high magnitude of plasticity of invasive *A*. *negundo* seedlings to variation in nutrient availability. Future studies should however test in the native range the response of native and invasive genotypes sampled at broader scales to a combination of abiotic factors in order to test more effectively both the importance of evolved *versus* pre-adapted plasticity and increases in competitive ability of invasive species.

## Supporting Information

Table S1 **Mean ± SE for traits related to growth, gas exchange and leaf morphology, biomass and biomass allocation of eight native and eight invasive populations of** ***Acer negundo*** **grown along a nutrient gradient.** Sample sizes are *n* = 24 for growth traits, *n* = 4 for physiology traits and *n* = 6 for leaf morphology and biomass related traits. See text for definition of terms.
(DOCX)

Table S2 **Intraspecific comparisons of phenotypic plasticity in invasive plant species.** Summary of studies comparing phenotypic plasticity between native and invasive populations of exotic plant species in response to variation in environmental conditions. Plasticity was reported for various traits related to biomass (B), defense to herbivory (D), growth (G), leaf morphology (M), phenology (Pe), physiology (P) and reproduction (R).
(DOCX)
