# Supplementary material for: A Test for Pre-Adapted Phenotypic Plasticity in the Invasive Tree Acer negundo L
Source: PLoS One. 2013 Sep 9;8(9):e74239. doi: 10.1371/journal.pone.0074239 (PMC3767822; doi:10.1371/journal.pone.0074239)
Supplement: Table S1 — Mean ± SE for traits related to growth, gas exchange and leaf morphology, biomass and biomass allocation of eight native and eight invasive populations of Acer negundo grown along a nutrient gradient. Sample sizes are n = 24 for growth traits, n = 4 for physiology traits and n = 6 for leaf morphology and biomass related traits. See text for definition of terms. (DOCX) [file pone.0074239.s001.docx]

**Table** **S1.** **Mean ± SE for traits related to growth, gas exchange and leaf morphology, biomass and biomass allocation of eight native and eight invasive populations of *Acer negundo* grown along a nutrient gradient.** Sample sizes are *n* = 24 for growth traits, *n* = 4 for physiology traits and *n* = 6 for leaf morphology and biomass related traits. See text for definition of terms.

| **Traits** | **Low nutrient level** | |  | **Medium nutrient level** | |  | **High nutrient level** | |
| --- | --- | --- | --- | --- | --- | --- | --- | --- |
|  | **Invasive** | **Native** |  | **Invasive** | **Native** |  | **Invasive** | **Native** |
| **Growth** |  |  |  |  |  |  |  |  |
| Height | 104.15 ± 2.24 | 92.51 ± 2.37 |  | 133.03 ± 2.55 | 111.58 ± 2.87 |  | 138.08 ± 2.89 | 112.45 ± 2.67 |
| Diameter | 9.69 ± 0.16 | 10.34 ± 0.16 |  | 11.35 ± 0.17 | 11.86 ± 0.18 |  | 12.06 ± 0.23 | 12.81 ± 2.22 |
| **Leaf traits** |  |  |  |  |  |  |  |  |
| *A*_area_ | 2.90 ± 0.17 | 3.00 ± 0.24 |  | 5.30 ± 0.33 | 5.38 ± 0.35 |  | 6.48 ± 0.44 | 6.53 ± 0.41 |
| *A*_mass_ | 0.11 ± 0.01 | 0.11 ± 0.01 |  | 0.18 ± 0.01 | 0.18 ± 0.02 |  | 0.21 ± 0.01 | 0.20 ± 0.01 |
| N_area_ | 0.38 ± 0.01 | 0.47 ± 0.02 |  | 0.62 ± 0.03 | 0.73 ± 0.03 |  | 1.10 ± 0.04 | 1.32 ± 0.06 |
| N_mass_ | 1.42 ± 0.07 | 1.57 ± 0.09 |  | 2.08 ± 0.08 | 2.34 ± 0.11 |  | 3.64 ± 0.07 | 3.87 ± 0.06 |
| PNUE | 7.64 ± 0.55 | 6.61 ± 0.54 |  | 9.10 ± 0.53 | 8.18 ± 0.70 |  | 5.83 ± 0.30 | 5.07 ± 0.27 |
| LMA | 28.62 ± 0.98 | 32.24 ± 1.35 |  | 30.63 ± 1.07 | 33.41 ± 1.40 |  | 30.28 ± 1.13 | 34.23 ± 1.38 |
| L_s_ | 34.32 ± 2.03 | 44.55 ± 2.45 |  | 39.02 ± 1.48 | 49.09 ± 2.58 |  | 47.92 ± 1.95 | 54.61 ± 2.23 |
| **Biomass** |  |  |  |  |  |  |  |  |
| *W*_l_ | 4.31 ± 0.46 | 3.96 ± 0.34 |  | 7.27 ± 0.48 | 7.35 ± 0.52 |  | 9.21 ± 0.74 | 8.90 ± 0.60 |
| *W*_s_ | 19.48 ± 2.05 | 15.34 ± 1.39 |  | 28.54 ± 2.09 | 25.89 ± 1.93 |  | 30.63 ± 2.91 | 29.86 ± 2.18 |
| *W*_r_ | 7.78 ± 0.81 | 10.29 ± 0.75 |  | 11.17 ± 1.04 | 14.41 ± 1.19 |  | 10.76 ± 1.08 | 14.63 ± 1.04 |
| *W*_t_ | 31.58 ± 3.22 | 29.71 ± 2.43 |  | 47.45 ± 3.41 | 47.43 ± 3.44 |  | 50.61 ± 4.62 | 53.39 ± 3.71 |
| *W*_a_ | 23.80 ± 2.47 | 19.30 ± 1.68 |  | 35.81 ± 2.53 | 33.24 ± 2.39 |  | 39.85 ± 3.59 | 38.76 ± 2.74 |
| *A*_l_ | 0.144 ± 0.015 | 0.128 ± 0.012 |  | 0.240 ± 0.015 | 0.217 ± 0.014 |  | 0.292 ± 0.021 | 0.253 ± 0.016 |
| **Biomass allocation** |  |  |  |  |  |  |  |  |
| RSR | 0.370 ± 0.022 | 0.580 ± 0.022 |  | 0.306 ± 0.017 | 0.455 ± 0.022 |  | 0.269 ± 0.010 | 0.401 ± 0.016 |
| LWR | 0.137 ± 0.005 | 0.134 ± 0.006 |  | 0.169 ± 0.006 | 0.162 ± 0.006 |  | 0.197 ± 0.007 | 0.174 ± 0.005 |
| SWR | 0.600 ± 0.010 | 0.504 ± 0.010 |  | 0.602 ± 0.008 | 0.532 ± 0.010 |  | 0.594 ± 0.006 | 0.544 ± 0.008 |
| RWR | 0.262 ± 0.010 | 0.362 ± 0.009 |  | 0.228 ± 0.010 | 0.305 ± 0.011 |  | 0.210 ± 0.006 | 0.282 ± 0.007 |
| LAR | 0.0051 ± 0.0003 | 0.0046 ± 0.0003 |  | 0.0061 ± 0.0005 | 0.0055 ± 0.0004 |  | 0.0072 ± 0.0004 | 0.0057 ± 0.0004 |
