# Supplementary material for: A Test for Pre-Adapted Phenotypic Plasticity in the Invasive Tree Acer negundo L
Source: PLoS One. 2013 Sep 9;8(9):e74239. doi: 10.1371/journal.pone.0074239 (PMC3767822; doi:10.1371/journal.pone.0074239)
Supplement: Table S2 — Intraspecific comparisons of phenotypic plasticity in invasive plant species. Summary of studies comparing phenotypic plasticity between native and invasive populations of exotic plant species in response to variation in environmental conditions. Plasticity was reported for various traits related to biomass (B), defense to herbivory (D), growth (G), leaf morphology (M), phenology (Pe), physiology (P) and reproduction (R). (DOCX) [file pone.0074239.s002.docx]

**Table S2.** **Intraspecific comparisons of phenotypic plasticity in invasive plant species.** Summary of studies comparing phenotypic plasticity between native and invasive populations of exotic plant species in response to variation in environmental conditions. Plasticity was reported for various traits related to biomass (B), defense to herbivory (D), growth (G), leaf morphology (M), phenology (Pe), physiology (P) and reproduction (R).

| **Invasive species** | **Functional group** | **Abiotic factors** | **Traits** | **Plasticity** | **Conclusion** | **References** |
| --- | --- | --- | --- | --- | --- | --- |
| *Alliaria petiolata* | Biennial forb | Nutrient, water | G, P | Inv = Nat | No post-introduction evolution | [1] |
| *Centaurea stoebe* | Perennial forb | Nutrient, water, site | G, M, P, R | Inv = Nat | Pre-adaptation | [2] |
| *Ceratophyllum demersum* | Perennial forb (aquatic) | Temperature | G, M, P | Inv > Nat | Post-introduction evolution | [3,4] |
| *Clidemia hirta* | Perennial shrub | Light | B, G, P | Inv = Nat | No genetic shift | [5] |
| *Cynoglossum officinale* | Biennial forb | Site | G, R | Inv > Nat | Founder effects | [6] |
|  |  | Nutrient | G, R | Inv = Nat | Founder effects |  |
| *Eupatorium adenophorum* | Perennial forb | CO_2_ | B, G | Inv = Nat | - | [7] |
| *Hypericum perforatum* | Perennial forb | Site | D | Inv = Nat | - | [8] |
| *Lythrum salicaria* | Perennial forb | Nutrient | B, R | Inv > Nat | - | [9] |
|  |  | Nutrient, water | B, G | Inv > Nat | Post-introduction evolution | [10] |
| *Melaleuca quinquenervia* | Tree | pH | G | Inv > Nat | - | [11] |
| *Microstegium vimineum* | Annual grass | Light, site | B, G, M | Inv = Nat | - | [12,13] |
| *Mimulus guttatus* | Annual forb | Water | G, Pe, R | Inv = Nat | No selection | [14] |
| *Phalaris arundinacea* | Perennial grass | Soil moisture | G, M | Inv > Nat | Post-introduction evolution | [15] |
| *Plantago lanceolata* | Perennial forb | Temperature | R | Inv = Nat | - | [16] |
| *Senecio inaequidens* | Perennial shrub | Nutrient | B | Inv > Nat | Post-introduction evolution | [17] |
| *Senecio pterophorus* | Perennial shrub | Disturbance, water | B, M, R | Inv > Nat | Selection | [18] |
| *Spartina alterniflora* | Perennial grass | Nutrient | B, G, M | Inv > Nat | Post-introduction evolution | [19] |
|  |  | Nutrient | P | Inv = Nat | - |  |
| *Taraxacum officinale* | Perennial forb | Water | B, M, P | Inv < Nat | Local adaptation | [20] |
|  |  | Water | B | Inv < Nat | - | [21] |
|  |  | Nutrient | B | Inv = Nat | - |  |
| *Triadica sebifera* | Tree | Light | B, M | Inv > Nat | Post-introduction evolution | [22] |
|  |  | Water | B | Inv = Nat | Pre-adaptation |  |

Inv: invasive populations, Nat: native populations, Inv = Nat: no difference in plasticity between native and invasive populations, Inv > Nat: invasive populations exhibit greater phenotypic plasticity than native populations, Inv < Nat: invasive populations exhibit lower phenotypic plasticity than native populations.

Site factor represents the effect of common gardens.

**References**

1. Hillstrom C, Cipollini D (2011) Variation in phenotypic plasticity among native and invasive populations of *Alliaria petiolata*. Int J Plant Sci 172: 763-772.
2. Hahn MA, van Kleunen M, Muller-Scharer H (2012) Increased phenotypic plasticity to climate may have boosted the invasion success of polyploid *Centaurea stoebe*. PLoS ONE 7(11): e50284. doi:10.1371/journal.pone.0050284.
3. Hyldgaard B, Brix H (2012) Intraspecies differences in phenotypic plasticity: Invasive versus non-invasive populations of *Ceratophyllum demersum*. Aquat Bot 97: 49-56.
4. Hyldgaard B, Sorrell B, Olesen B, Riis T, Brix H (2012) Geographically distinct *Ceratophyllum demersum* populations differ in growth, photosynthetic responses and phenotypic plasticity to nitrogen availability. Funct Plant Biol 39: 774-783.
5. DeWalt SJ, Denslow JS, Hamrick JL (2004) Biomass allocation, growth, and photosynthesis of genotypes from native and introduced ranges of the tropical shrub *Clidemia hirta*. Oecologia 138: 521-531.
6. Williams JL, Auge H, Maron JL (2008) Different gardens, different results: native and introduced populations exhibit contrasting phenotypes across common gardens. Oecologia 157: 239-248.
7. Lei YB, Feng YL, Zheng YL, Wang RF, Gong HD, Zhang YP (2011) Innate and evolutionarily increased advantages of invasive *Eupatorium adenophorum* over native *E. japonicum* under ambient and doubled atmospheric CO2 concentrations. Biol Invasions 13: 2703-2714.
8. Maron JL, Vila M, Arnason J (2004) Loss of enemy resistance among introduced populations of St. John's Wort (*Hypericum perforatum*). Ecology 85: 3243-3253.
9. Chun YJ (2011) Phenotypic plasticity of introduced versus native purple loosestrife: univariate and multivariate reaction norm approaches. Biol Invasions 13: 819-829.
10. Chun YJ, Collyer ML, Moloney KA, Nason JD (2007) Phenotypic plasticity of native vs. invasive purple loosestrife: A two-state multivariate approach. Ecology 88: 1499-1512.
11. Kaufman SR, Smouse PE (2001) Comparing indigenous and introduced populations of *Melaleuca quinquenervia* (Cav.) Blake: response of seedlings to water and pH levels. Oecologia 127: 487-494.
12. Flory SL, Long FR, Clay K (2011) Invasive *Microstegium* populations consistently outperform native range populations across diverse environments. Ecology 92: 2248-2257.
13. Flory SL, Long FR, Clay K (2011) Greater performance of introduced vs. native range populations of *Microstegium vimineum* across different light environments. Basic Appl Ecol 12: 350-359.
14. van Kleunen M, Fischer M (2008) Adaptive rather than non-adaptive evolution of *Mimulus guttatus* in its invasive range. Basic Appl Ecol 9: 213-223.
15. Lavergne S, Molofsky J (2007) Increased genetic variation and evolutionary potential drive the success of an invasive grass. P Nat Ac S USA 104: 3883-3888.
16. Alexander JM, van Kleunen M, Ghezzi R, Edwards PJ (2012) Different genetic clines in response to temperature across the native and introduced ranges of a global plant invader. J Ecol 100: 771-781.
17. Bossdorf O, Lipowsky A, Prati D (2008) Selection of preadapted populations allowed *Senecio inaequidens* to invade Central Europe. Divers Distrib 14: 676-685.
18. Caño L, Escarre J, Fleck I, Blanco-Moreno JM, Sans FX (2008) Increased fitness and plasticity of an invasive species in its introduced range: a study using *Senecio pterophorus*. J Ecol 96: 468-476.
19. Qing H, Yao YH, Xiao Y, Hu FQ, Sun YX, Zhou CF, An SQ (2011) Invasive and native tall forms of *Spartina alterniflora* respond differently to nitrogen availability. Acta Oecol 37: 23-30.
20. Molina-Montenegro MA, Quiroz CL, Torres-Diaz C, Atala C (2011) Functional differences in response to drought in the invasive *Taraxacum officinale* from native and introduced alpine habitat ranges. Plant Ecol Divers 4: 37-44.
21. Quiroz CL, Choler P, Baptist F, Gonzalez-Teuber M, Molina-Montenegro MA, Cavieres LA (2009) Alpine dandelions originated in the native and introduced range differ in their responses to environmental constraints. Ecol Res 24: 175-183.
22. Zou JW, Rogers WE, Siemann E (2009) Plasticity of *Sapium sebiferum* seedling growth to light and water resources: Inter- and intraspecific comparisons. Basic Appl Ecol 10: 79-88.
